# Genetic diversity and phylogeographic patterns of the dioecious palm *Chamaedorea tepejilote* (Arecaceae) in Costa Rica: the role of mountain ranges and possible refugia

**DOI:** 10.1093/aobpla/plac060

**Published:** 2022-12-17

**Authors:** Eric J Fuchs, Alfredo Cascante-Marín, Ruth Madrigal-Brenes, Mauricio Quesada

**Affiliations:** Escuela de Biología, Universidad de Costa Rica, Ciudad Universitaria Rodrigo Facio, San Pedro Montes de Oca 11501-2060, Costa Rica; Laboratorio Nacional de Análisis y Síntesis Ecológica, Escuela Nacional de Estudios Superiores Unidad Morelia, Universidad Nacional Autónoma de México, Morelia, Michoacán, 58190, México; Laboratorio Binacional de Análisis y Síntesis Ecológica, UNAM-UCR, México–Costa Rica; Escuela de Biología, Universidad de Costa Rica, Ciudad Universitaria Rodrigo Facio, San Pedro Montes de Oca 11501-2060, Costa Rica; Laboratorio Binacional de Análisis y Síntesis Ecológica, UNAM-UCR, México–Costa Rica; Escuela de Biología, Universidad de Costa Rica, Ciudad Universitaria Rodrigo Facio, San Pedro Montes de Oca 11501-2060, Costa Rica; Laboratorio Nacional de Análisis y Síntesis Ecológica, Escuela Nacional de Estudios Superiores Unidad Morelia, Universidad Nacional Autónoma de México, Morelia, Michoacán, 58190, México; Laboratorio Binacional de Análisis y Síntesis Ecológica, UNAM-UCR, México–Costa Rica; Laboratorio Nacional de Análisis y Síntesis Ecológica, Escuela Nacional de Estudios Superiores Unidad Morelia, Universidad Nacional Autónoma de México, Morelia, Michoacán, 58190, México; Laboratorio Binacional de Análisis y Síntesis Ecológica, UNAM-UCR, México–Costa Rica; Instituto de Investigaciones en Ecosistemas y Sustentabilidad, Universidad Nacional Autónoma de México, Morelia, Michoacán, 58190, México

**Keywords:** Chloroplast DNA, gene flow, Lower Central America, microsatellites, Talamanca Mountain range

## Abstract

Gene flow connects populations and is necessary to sustain effective population sizes, and genetic diversity. In the Lower Central American (LCA) region, the complex topographic and climatic history have produced a wide variety of habitats resulting in high biodiversity. Phylogeographic studies of plants from this area are scarce, and to date none have been conducted on palms. We used SSR and chloroplast DNA (cpDNA) markers to study the genetic diversity and structure of populations of the understory palm *Chamaedorea tepejilote* in Costa Rica. We found that populations of *C. tepejilote* have moderate to high nuclear simple sequence repeat (SSR) genetic diversity, likely due to large population sizes and its outcrossing mating system. Habitat loss and fragmentation may have contributed to increased genetic structure within slopes. High-elevation mountain ranges appeared to be a significant barrier for gene flow among populations in the Caribbean and Pacific slopes; however, ranges are permeable through low-elevation passes. In contrast, most populations had a single distinct cpDNA haplotype, supporting the hypothesis of several isolated populations that experienced decline that likely resulted in eroded cytoplasmic genetic diversity within populations. The haplotype network and Bayesian analysis linked populations in the Caribbean and the southern Pacific coast, suggesting that gene flow between Pacific and Caribbean populations may have occurred through the southern extreme of the Talamanca Mountain range in Panama, a colonization pathway not previously suggested for LCA plants. This is one of the first phylogeographic studies conducted on tropical palms in the LCA region and the first in the genus *Chamaedorea*, which sheds light on possible gene flow and dispersal patterns of *C. tepejilote* in Costa Rica. Our results also highlight the importance of mountain ranges on shaping gene flow patterns of Neotropical plants.

## Teaser

This is one of the first phylogeographic studies on tropical palms in Lower Central America (LCA) and the first in the genus *Chamaedorea*. It gives insight on gene flow and dispersal patterns of *Chamaedorea tepejilote*in Costa Rica. *Chamaedorea tepejilote*populations contain moderate to high nuclear genetic diversity and very minimal chloroplast DNA diversity. High-elevation mountain ranges appear to be a barrier for gene flow between populations on the Caribbean and Pacific slopes of Costa Rica, although low-elevation passages are permeable. Gene flow between Pacific and Caribbean populations may have occurred through Panama, a colonization pathway not previously considered for LCA plants.

## Introduction

The importance of gene flow on the magnitude and distribution of genetic diversity of plant species is widely recognized (Frankham 1995, 2005; Couvet 2002). Gene flow connects populations over long distances and increases effective population sizes, ameliorating the eroding effects of genetic drift on diversity (Booy *et al.* 2000; Couvet 2002). Gene flow also maintains genetic variability within populations and understanding how this genetic diversity is geographically structured provides valuable information on how different landscape components such as habitat availability (Dick and Heuertz 2008), habitat fragmentation (Fuchs *et al.* 2003; Fuchs and Hamrick 2011; Aguilar *et al.* 2019), urban development (Delaney *et al.* 2010), and topographic barriers (Antonelli 2017) shape gene dispersal patterns among plant populations. Gene flow patterns directly influence effective population sizes, the connectivity of populations and the likelihood of local extinction (Lande 1988; Schemske *et al.* 1994; Hanski and Gilpin 1997). Estimates of historical and contemporary levels of gene flow are essential for designing conservation strategies that maximize diversity and connectivity among populations (Ellstrand 1992; Newton *et al.* 1999).

The Talamanca Mountain range in the Lower Central American region (LCA) is a biologically and topographically complex area and several studies recognize it as a world biodiversity hotspot for plants (Bagley *et al.* 2014; Brummitt *et al.* 2021). The biodiversity of this region increased after the formation of the Central American Isthmus (Gabb 2007; Montes *et al.* 2012; Bacon *et al.* 2015; O’Dea *et al.* 2016) which served as a land bridge and allowed for the exchange of the North and South American biotas (Bagley *et al.* 2014). Many plant species of southern and northern origin of the continent have only recently colonized this area (Gentry 1982), and their populations may have experienced demographic shifts during the climatic fluctuations of the late Pleistocene and early Holocene (Hooghiemstra *et al.* 1992). The uplifting of the Talamanca Mountain range around 15 My ago, the highest elevation (3860 m) of the Central American Isthmus, created a complex topography with abrupt elevation, humidity and temperature gradients, which resulted in high environmental heterogeneity and a large availability of ecological niches that likely promoted speciation. Furthermore, the Talamanca Mountain range extends from the south-eastern to north-western part of the LCA separating the Pacific and Caribbean lowlands; this also suggests the potential role of the Talamanca Mountain range as a barrier to gene dispersal between populations on both slopes. Therefore, the LCA provides a good opportunity to study genetic structure and phylogeographic patterns for plant populations aiming to identify patterns of species colonization and the factors that influence contemporary gene flow and population genetic structuring, as well as the resilience of plant populations to future climate changes.

The few phylogeographic studies conducted on plants from the LCA have primarily focused on tree species (but see Trapnell *et al.* 2019) and suggest that many populations survived glaciations in refugia. According to multiple authors (Brown 1987; Poelchau and Hamrick 2013), species may have survived in lowland refugia due to increased precipitation and more land cover during glacial eras; these refugia likely supported large populations and high genetic diversity. Conversely, species may have retreated into mid-elevation refugia, leading to a decline in population sizes and insularization (Brown 1987; Dick and Heuertz 2008; Jones *et al.* 2013). This likely resulted in a reduction in genetic diversity within refugia and an increase in genetic structure. A common denominator of most studies conducted on tropical trees is that mountain ranges play a predominant role in limiting gene flow and structuring genetic diversity (Chase *et al.* 1995; Aide and Rivera 1998; Cavers *et al.* 2003; Dick *et al.* 2003; Novick *et al.* 2003; Dick and Heuertz 2008; Poelchau and Hamrick 2011). In the tropics, strong habitat selection restricts the ability of plants to disperse to high mountains (Janzen 1967). This creates a significant physiological barrier for gene flow (Ghalambor *et al.* 2006), thereby structuring lineages across mountain ranges (Avise 2000).

Because the genetic structure of plant populations is strongly dependent on life history traits (Hamrick and Loveless 1987; Hamrick and Godt 1996; Poelchau and Hamrick 2011), more studies on different plant taxa are needed to understand the factors that shape the genetic structure and phylogeographic patterns of plants from the LCA. Palms are distinctive elements of tropical forests (Henderson *et al.* 2019) and are regarded as keystone resources for tropical habitat biota (Terborgh 1986). Palms are also a locally abundant plant guild, and their generation time is shorter compared to trees (Oyama 1990), so they are likely to respond differently to climate and landscape changes. Studies on the geographic distribution of genetic diversity of palms have been predominantly conducted in South America (Souza and Martins 2004; Trénel *et al.* 2008; de Lima *et al.* 2014; Escobar *et al.* 2018, 2021; Souza *et al.* 2018) and these studies have shown that genetic structure is strongly influenced by habitat availability, dispersal limitation and topographic barriers. The genus *Chamaedorea* is the largest palm genus in the Neotropics (Goaverts *et al.* 2020) with more than 100 dioecious species. The Talamanca Mountain range in LCA (Costa Rica and Western Panama) is believed to be a secondary centre of diversity for the genus, while the primary centre is located in southern Mexico and Guatemala in the northern part of Central America (Hodel 1992; Henderson *et al.* 2019). To date, no studies have been conducted on the genetic diversity or phylogeography of *Chamaedorea* palm species in the LCA.

The main objective of this study is to analyse the genetic diversity and structure of populations of *Chamaedorea tepejilote* in Costa Rica, using nuclear microsatellites (SSRs), and describe phylogeographic patterns using maternally inherited chloroplast DNA (cpDNA) markers. We propose the following hypotheses: (i) previous research on *C. tepejilote* populations in southern Mexico has demonstrated that gene flow frequently occurs over great distances (Luna *et al.* 2005, 2007); therefore, we hypothesize high levels of genetic diversity and limited genetic structure among populations. However, (ii) we expect that the Talamanca Mountain range acts as the main barrier to gene flow between populations from the Pacific and Caribbean slopes. (iii) We hypothesize that during the Pleistocene glaciations, Costa Rican populations of *C. tepejilote* likely maintained large populations in lowland forest refugia, as suggested by Poelchau and Hamrick (2011). Consequently, we hypothesize relatively high haplotype diversity and low phylogeographic structure in lowland populations, with structure determined primarily by high mountain ranges acting as barriers to gene flow. In contrast, if populations retreated into multiple refugia, we expect to find low haplotypic diversity within populations and high levels of genetic structure due to isolation and drift (Brown 1987; Dick and Heuertz 2008; Jones *et al.* 2013).

## Methods

### Study species

*Chamaedorea tepejilote* is a dioecious palm species distributed from Mexico to Colombia. This palm develops a single erect stem 5-m high with three to six pinnate leaves per stem, branched, racemose inflorescences with several rachillae, small (<5 mm), greenish to yellow, scented flowers, dry-powdery pollen and black fruits at maturity (Hodel 1992). It is an ambophylous species (Ríos *et al.* 2013), pollinated by thrips (Thysanoptera) and wind, with fruits dispersed by birds and mammals (Hodel 1992). The centre of origin of *C. tepejilote* is believed to be Mexico (Hodel 1992). Populations of *C. tepejilote* in Mexico have been shown to have a stem length growth of 6–12 cm per year, with 2.3 new leaves produced each year (Oyama 1990). Although generation time is still undetermined, plants below 1 m in height are rarely reproductive. *Chamaedorea tepejilote* is a common component of the understory vegetation and is frequently found along streams or riparian habitats within mature forests, from sea level up to 1600 m a.s.l. (Mont *et al.* 1994). This species has economic importance due to its ornamental and medicinal properties, and male inflorescences are consumed as food in some Central American countries (Mont *et al.* 1994; Pérez *et al.* 2008; Robles and Carranza 2013). Deforestation and urbanization have likely reduced populations found within natural habitats, reducing effective population sizes, and increasing isolation among populations (Luna *et al.* 2007).

### Population sampling

**Figure 1.**
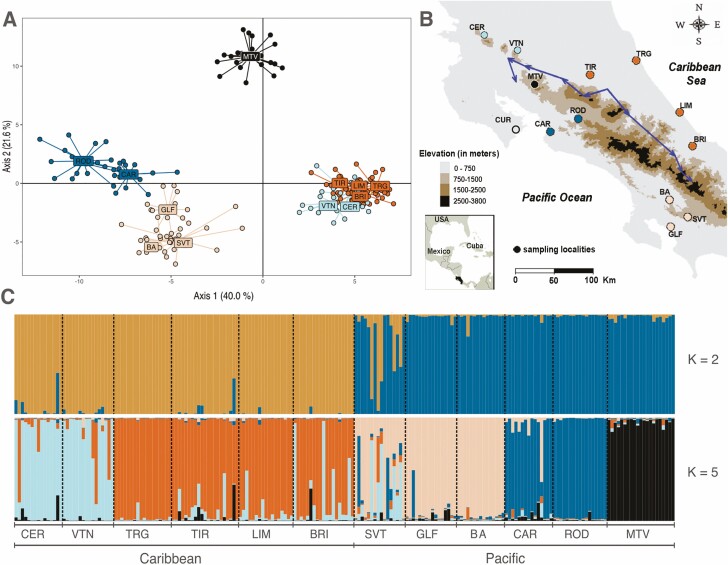
(A) Discriminant analysis of principal components for 12 populations of Chamaedorea tepejilote (Arecaceae) in Costa Rica. Axes show the percentage of the variation explained. Each population is coded based on clusters defined by STRUCTURE *K* = 5 (C). Centroid labels have a small nudge to improve readability. (B) Map of Costa Rica showing populations (dots) of *C. tepejilote* (Arecaceae) sampled and the most likely barrier found by the Monmonier algorithm (arrows). CUR population (clear dot) had only three samples and could not be included in the barrier calculation. (C) Bayesian assignment performed by STRUCTURE for 12 populations in Costa Rica for *K* = 2 and *K* = 5 clusters. Each vertical bar represents an individual and is divided proportionally to the probability of assignment of each individual to each genetic cluster. Refer to Table 1 for population codes. Shades in panels A and B are the same as in *K* = 5..

We collected leaf tissue from 15–22 individuals in each of 13 populations in Costa Rica (Table 1; Fig. 1B). We selected sampling sites in mature continuous forests that had not been disturbed for at least 40 years and had at least 30 adult *C. tepejilote*. Most populations were located within National Parks or Forest Reserves and had reproductive individuals, as we always found seedlings and juveniles during collections. Young leaves were selected from mature individuals that were separated by at least 10 m from each other. Leaves were finely cut and placed in 50-mL Falcon tubes with silica powder (Sigma-Aldrich; 70-230 mesh, 63–200 μm). Silica was replaced at least twice before DNA extraction to ensure proper tissue dehydration. Nuclear and plastid DNA were extracted using the modified CTAB protocol from Doyle and Doyle (1990).

**Table 1.** Genetic diversity estimates for 13 *Chamaedorea tepejilote* (Arecaceae) populations in Costa Rica. *N*: sample size; Ar: allelic richness; *H*_O_: observed heterozygosity; *H*_E_: expected heterozygosity; *F*_IS_: inbreeding coefficient. Mean values ± (SE).

Population	Code	Latitude/longitude	Elevation (m)	*N*	Ar	*H*_O_	*H*_E_	*F*_IS_
Buenos Aires	BA	8.988611 –83.19302	180	15	6.20 (0.95)	0.766 (0.05)	0.670 (0.06)	–0.169*
Bribri	BRI	9.609722 –82.90139	55	19	4.86 (1.01)	0.521 (0.10)	0.556 (0.09)	0.0290
Carara	CAR	9.775400 –84.60507	28	15	7.00 (0.95)	0.766 (0.06)	0.716 (0.05)	–0.076
Cervantes	CER	10.873333 –85.39284	666	15	6.00 (0.90)	0.640 (0.10)	0.634 (0.08)	0.080
Golfito	GLF	8.656953 –83.17691	96	16	6.19 (0.86)	0.737 (0.07)	0.706 (0.05)	–0.058
Limón	LIM	9.983333 –83.06222	46	17	5.13 (1.06)	0.635 (0.11)	0.573 (0.10)	–0.108
Monteverde	MTV	10.308333 –84.79417	1500	22	6.32 (0.72)	0.706 (0.06)	0.725 (0.04)	0.030
Rodeo	ROD	9.907316 –84.27458	1008	17	5.23 (0.88)	0.629 (0.07)	0.641 (0.06)	0.025
San Vito	SVT	8.785277 –82.95917	1210	16	7.35 (0.87)	0.675 (0.06)	0.748 (0.05)	0.103
Tirimbina	TIR	10.417297 –82.95917	200	21	5.94 (0.98)	0.600 (0.10)	0.605 (0.09)	0.048
Tortuguero	TRG	10.574871 –83.57705	17	18	4.48 (0.83)	0.583 (0.11)	0.553 (0.09)	–0.054
Volcán Tenorio	VTN	10.700555 –84.99694	770	16	5.97 (0.94)	0.593 (0.09)	0.613 (0.08)	–0.001

*Significant *P* < 0.01 based on 1000 bootstrap values.

### Microsatellites

To analyse the genetic diversity and structure of *C. tepejilote* we used 10 SSR loci developed exclusively for *C. tepejilote* and used the sequences and PCR amplification protocols detailed in Fuchs *et al.* (2020). Amplification products were visualized in an ABI-3500 Sanger sequencer at Escuela de Biología at Universidad de Costa Rica, and genotyped using GeneMarker version 2.4.2 in the Laboratorio Nacional de Análisis y Síntesis Ecológica (LANASE) at Universidad Nacional Autónoma de México (UNAM) visual computer cluster. We tested for the presence of null alleles or allelic dropout with Microchecker v2.2.3.

Genetic diversity and structure statistics were estimated using the *adegenet* (Jombart 2008; Jombart and Ahmed 2011), *poppr* (Kamvar *et al.* 2014), *mmod* (Winter 2012) and *hierfstat* (Goudet 2005) libraries from the R statistical language (R Development Core Team 2020). We only found three *C. tepejilote* plants at CUR; therefore, this population was excluded from microsatellite analyses, since allele frequencies are not accurately estimated with only three individuals. We estimated allelic and heterozygosity diversity measures and used Nei’s *G*_ST_ to estimate genetic structure among populations. To test the hypothesis that mountain ranges play a key role in shaping population structure, we performed an analysis of molecular variance (AMOVA), using Pacific and Caribbean slopes as the regional strata. The AMOVA analysis implemented in the *poppr* package was used, using 9999 permutations to test the significance of phi-statistics. Pairwise *G*_ST_ values were used to construct an unrooted tree based on the neighbour-joining (NJ) algorithm using the *ape* (Paradis and Schliep 2019) library in R. Isolation by distance (IBD) was tested using a Mantel correlation between pairwise *G*_ST_ and Euclidean distances (5000 permutations). We assessed the significance of all statistics by bootstrapping samples 1000 times among populations. To identify possible geographic barriers to gene flow, we used the Monmonier algorithm (Monmonier 1973) in the *adegenet* package using Nei’s pairwise *G*_ST_ values and default thresholds. We also visualized similarity among individuals using the discriminant analysis of principal components (DAPC) implemented in *adegenet*. We retained 70 PCs as suggested by cross-validation with 999 replicates.

The Bayesian clustering algorithm implemented in STRUCTURE (Falush *et al.* 2003) was also used to determine the best configuration of samples into *K* clusters based on similarity in allele frequencies, and possible admixture among clusters. We used the admixture model with correlated allele frequencies, with 50 000 MCMC chains and a *burnin* of 10 000 chains. We estimated the likelihood of each configuration for *K* between 2 and 8, using 15 replicates for each *K* value. *StructureHarvester* v0.6.94 (Earl and VonHoldt 2012) was used to determine the most likely number of *K* clusters. As recommended by Janes *et al.* (2017) and the STRUCTURE manual (Pritchard and Wen 2003), we also plotted the average across all iterations of the estimated likelihood of *K* for each value of *K* and selected the optimal *K* at the point at which the plot curvature plateaus. We used CLUMPP version 1.1.2 to process all the runs for the most likely *K* using the greedy algorithm and produce assignment bar graphs for individuals and populations using the *pophelper* library (Francis 2017) in R.

### Chloroplast DNA

To describe the phylogeographic structure of *C. tepejilote*, we used two cpDNA sequences as plastid markers, the *rps16–trnQ* and the *ndhG–ndhI* intergenic spacers using primers developed by Scarcelli *et al.* (2011). Sequences were amplified using the TopTaq Master Mix Kit (Qiagen, North Carolina [NC], USA), according to manufacturer’s instructions. Reactions were conducted in 12.5 mL with 25 ng of DNA. The initial denaturation (94 °C/3 min) was followed by 35 cycles of denaturation (94 °C/30 s), annealing (58 °C for *ndhG–ndhI* and 62 °C for *rps16–trnQ*/30 s), an elongation period of 1 min at 72 °C and a final elongation step (72 °C/10 min). Amplification was checked on agarose gels. PCR products were cleaned and sequenced in forward and reverse directions at Macrogen (Seoul, Korea).

Forward and reverse sequences were assembled using Geneious X in the graphic cluster at LANASE. Alignments were checked by hand to resolve discrepancies and consensus sequences were extracted. We aligned sequences separately using MAFFT 7.475 and trimmed sequences of both genes, so they would be of the same size. We then concatenated sequences for *rps16–trnQ* and *ndhG–ndhI* and realigned them using MAFFT again. Sequences were imported into DNAsp version 6.12.01 and molecular and haplotype diversity was estimated. We also extracted haplotypes into a new Nexus file, which was imported into PopArt v. 1.7 to construct a median-joining haplotype network and plot haplotype frequency onto a map of Costa Rica. We also used DNAsp version 6.12.01 to estimate within and among population divergence using the *G*_ST_ estimate of Nei and the *N*_ST_ estimate proposed by Lynch and Crease (1990) that take allele origin into account. We also estimated Tajima’s *D* and Fu’s *F*, to test for neutrality and demographic change. Haplotypes were also analysed by means of an AMOVA using Arlequin 3.5 (Excoffier and Lischer 2010) to test if structure was primarily determined by mountain ranges. We compared haplotypes among Pacific and Caribbean slopes $\phi_{CT}$) and the proportion of genetic variability among populations $\phi_{ST}$). We used 35 000 permutations and the proportion of differences as a distance measure in Arlequin.

We used the Bayesian clustering algorithm implemented in BAPS 6 (Corander *et al.* 2004) to infer the genetic structure from cpDNA haplotypes. We used the linked loci procedure with default values to test for different configurations from *K* = 1 to *K* = 20. We performed an admixture analysis on the results, using five as the minimum population size, and 5000 iterations per reference.

### Ecological niche modelling

To determine biogeographic barriers of the study species according to topography, we used ecological niche modelling (ENM) as implemented in the *wallace* 1.9.0 library (Kass *et al.* 2020) in R. With *wallace* we downloaded all the locations of *C. tepejilote* from GBIF (www.gbif.org), filtered duplicate locations and registries without location information. We also eliminated records that were clearly outside the described distribution of the species and most likely represent registry errors (e.g. samples in the Atlantic or Africa). We also performed a spatial thinning to keep only samples that were separated by at least 5 km from each other. To determine the most suitable habitat we initially used the 19 environmental variables available from WorldClim v. 1.4 data set with a 30 arcmin resolution and eliminated variables 8, 9, 10 and 11 which were strongly correlated among them (*r* > 0.9). We modelled species distribution using the *Maxent* algorithm, which is based on presence–absence data (Phillips *et al.* 2006). We tested linear, quadratic and hinge feature class models, as well as regularization multipliers between 1 and 3, with a step value of 1 and included clamping in the model. *K*-fold cross-validation was used and data were randomly split into four groups to validate the model. We chose the model with the lowest omission rate of the training set and higher Area under the curve (AUC) values. Model suitability predictions were visualized using the *clog-clog* transformation.

## Results

### Genetic diversity and structure using SSRs

All 10 SSR loci were polymorphic with an average allelic richness of 5.80 ± 0.243 alleles. The mean observed and expected heterozygosities were *H*_O_ = 0.655 ± 0.071 and *H*_E_ = 0.645 ± 0.062, respectively. Diversity estimates were consistently high across all populations (*H*_E_ = 0.553–0.748; Table 1). Only the BA population had a significant excess of heterozygotes (*F*_IS_ = −0.169), while all other inbreeding coefficients were not statistically different from zero.

We found significant genetic structure among all populations $G_{ST}=0.144$, *P* < 0.001) using SSR markers. The AMOVA found significant genetic structure among Pacific and Caribbean slopes $\phi_{CT}=0.178$; *P* < 0.001; **see****Supporting Information—Table S1**), and significant differences in allele frequencies among populations within slopes $\phi_{SC}=0.191$; *P* < 0.001; **see****Supporting Information—Table S1**). We did not find significant evidence of IBD $r_{M}=0.040$, *P* = 0.333). If we estimated the distance between populations taking elevation into account, the Mantel correlation coefficient increased in magnitude, however it was still not statistically significant $r_{M}=0.222$, P=0.08).

Discriminant analysis of principal components grouped populations on the southern and central Pacific coast (ROD, CAR, GLF, BA, SVT), while all the Caribbean populations (TRG, TIR, VTN, CER, BRI, LIM) formed another group, and MTV as the most distant population on DAPC space (Fig. 1A), indicating greater differences in this population, but still linked to Pacific populations. StructureHarvester grouped individuals into K=2 clusters separating populations in the Pacific and Caribbean slopes (Fig. 1B), but the likelihood curve suggested *K* = 5 **[see****Supporting Information—Fig. S1****]**, which assigned individuals into a similar configuration as that suggested by DAPC and the NJ tree **[see****Supporting Information—Fig. S2****]**. The Monmonier’s algorithm found a barrier separating Pacific from Caribbean populations (Fig. 1C). The barrier found by the algorithm corresponds with the main mountain ranges that dissect the country into two different climatic and topographic regions.

### cpDNA diversity

**Figure 2.**
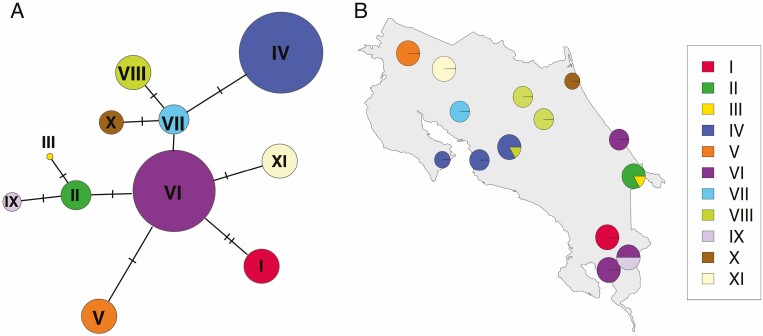
(A) Median-joining haplotype network for populations of *Chamaedorea tepejilote* (Arecaceae) in Costa Rica. Circle sizes reflect the frequency of the haplotype. (B) Map of Costa Rica showing the distribution of *C. tepejilote* haplotypes in each sampled population.

We successfully sequenced 70 individuals from 13 populations. Sequences for the *rps16–trnQ* and the *ndhG–ndhI* intergenic spacers were 902 bp and 1066 bp long, respectively. Concatenated sequences had 10 segregating sites and nucleotide diversity was π=0.00123±0.00006 and $\theta_{W}=2.075\pm 0.431$. All 70 sequences could be grouped into 11 different haplotypes, including gaps and *indels* as variants. Haplotype diversity or the probability that two randomly chosen molecules are different was h=0.887±0.018. Tajima’s *D* = 0.38517 (*P* > 0.10) and Fu’s *F* = 0.012 (*P* > 0.10), which suggests neutrality and no evidence of recent demographic changes. Nearly all populations were composed of a single haplotype (except in ROD and BRI) and the haplotype network (Fig. 2) shows that most haplotypes are separated by a single mutation, except for haplotype I, which is separated by two mutations from haplotype VI. Haplotypes IV and VI are the most common haplotypes. Haplotype IV is distributed among central Pacific populations (CAR, ROD and CUR), while haplotype VI is present in populations in the southern part of Costa Rica, on both the Pacific and Caribbean slopes. An interesting result of the network topology is that the two northern populations CER and VTN are linked to haplotype VI which is mostly found in the southern coast of Costa Rica. Haplotype genetic structure was significantly more pronounced $G_{ST}=0.9323$, P<0.001 and $N_{ST}=0.9697$, P<0.0001) than the genetic structure estimated using microsatellites. Genetic structure estimated using an AMOVA was also high and statistically significant $\Phi_{ST}=0.967$, P<0.0001), since very few haplotypes are shared among populations; however, we did not find significant differences in allele frequencies among populations in the Pacific and Caribbean slopes $\phi_{CT}=0.087$, P>0.05; **see****Supporting Information—Table S2**).

**Figure 3.**
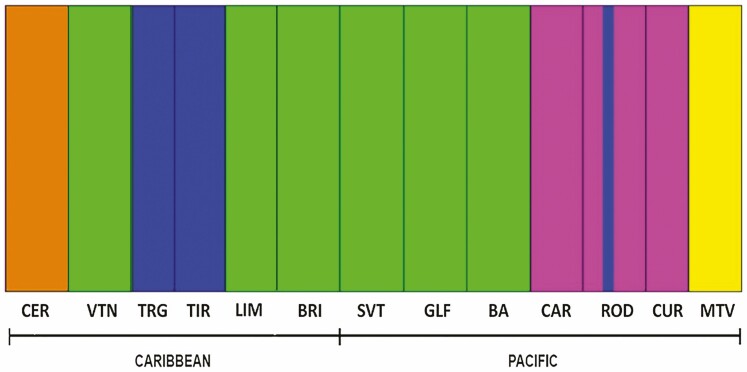
Population genetic structure based on cpDNA using Bayesian assignment inferred with BAPS (*K* = 5) for individuals of *Chamaedorea tepejilote* (Arecaceae) from 13 populations in Costa Rica.

BAPS found that K=5 is the most likely number of clusters to group haplotypes (Fig. 3). The program did not find evidence of admixture and all sequenced individuals were assigned to a single cluster. BAPS placed CER and MTV populations into separate clusters; while another cluster is composed of the northern Caribbean populations (TIR, TRG). Central Pacific populations CAR, CUR and ROD were clustered into another group, while the remaining populations are in a major cluster formed by populations from the southern Caribbean (LIM, BRI) and southern Pacific coast (SVT, GLF, BA) of the country (Fig. 3).

### Ecological niche modelling

**Figure 4.**
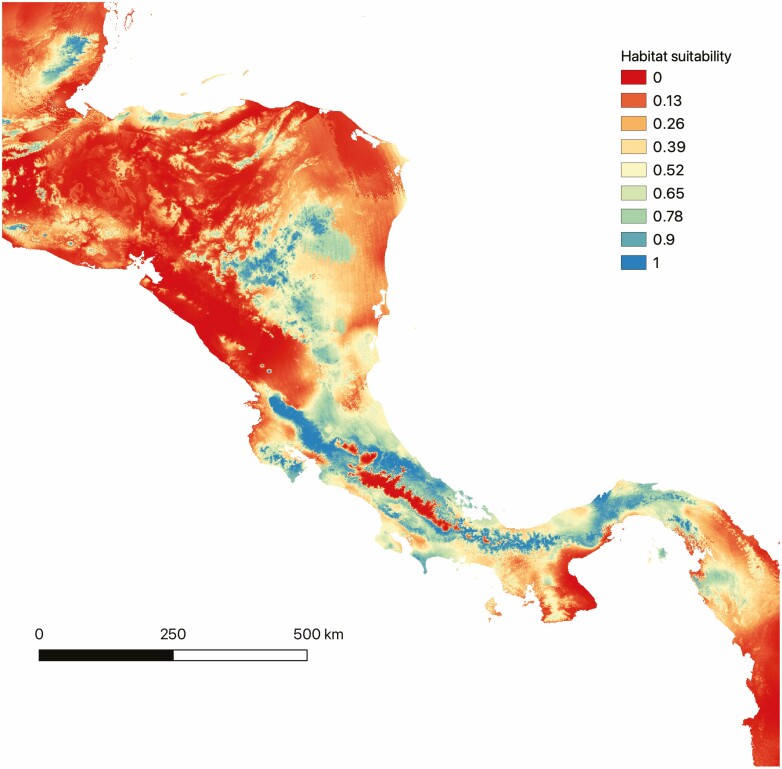
Ecological niche modelling predictions for habitat suitability of *C. tepejilote* (Arecaceae) in the LCA region, constructed using the GBIF occurrences and the Maxent algorithm. Cooler colours suggest higher habitat suitability.

The Hinge model had the highest AUC value (average AUC = 0.859). The predicted distribution model showed that *C. tepejilote* habitat suitability was lower in dry forest lowlands in the north-western pacific region of the country and at high elevations along the Talamanca Mountain range (Fig. 4). In Costa Rica, most suitable habitats for *C. tepejilote* are located at intermediate elevations, but humid lowlands are also apt for this palm species. This species is unable to inhabit at high elevations in montane forests; therefore, mountain ranges likely act as barriers for this species.

## Discussion

We found moderate to high levels of microsatellite genetic diversity in the palm *C. tepejilote*, as expected for a widely distributed and obligately outcrossing dioecious plant species. We found significant genetic structure among populations of this palm species in Costa Rica, with mountain ranges acting as predominant barriers to gene flow, likely due to the inability of palms to withstand environmental conditions at high elevations. In contrast, plastid markers had almost no within population genetic diversity but a significant phylogeographic structure, congruent with the hypothesis that populations may have resided into multiple refugia, experiencing isolation and drift. Our analyses revealed that haplotypes found on the southern Caribbean and southern Pacific coasts are closely related, indicating that gene flow may have occurred through the southernmost extent of the Talamanca Mountain range; a dispersal pattern not previously proposed for LCA plants. This is the first phylogeographic study on tropical palms in the LCA, a region with high palm diversity.

### Microsatellites

We found moderate to high levels of genetic diversity in *C. tepejilote*, using SSR markers, as we originally hypothesized for a widespread outcrossing species. Genetic diversity of palms has been predominantly studied in commercial or edible species such as oil-producing palms or date palms (e.g. Conte *et al.* 2003; Hayati *et al.* 2004; Huda *et al.* 2018; El Kadri *et al.* 2019; Gan *et al.* 2021). Although still relatively scant, most studies of genetic diversity on understory palms found comparable levels of genetic diversity, for example, Cibrian-Jaramillo *et al*. (2009) found for *C. ernesti-augusti* an average heterozygosity of *H*_E_ = 0.67 (CI 95 % ± 0.06) and *H*_O_ = 0.39 (CI 95 % ± 0.049); while Lanes *et al.* (2016), using nine microsatellite loci in *Acrocomia aculeata*, found average heterozygosity values (*H*_O_ = 0.527, *H*_E_ = 0.678) similar to those found in this study for *C. tepejilote*. The heterozygosity levels estimated in our study for *C. tepejilote* match those expected for a widespread outcrossing species and are higher than those observed for short-lived perennials (Nybom 2004). Previous studies conducted in Mexico and Belize in species from the genus *Chamaedorea* posit that large effective populations and ample levels of gene flow are the main causal factors for the observed levels of gene diversity (Luna *et al.* 2007; Cibrian-Jaramillo *et al.* 2009; Peñaloza-Ramírez *et al.* 2016). *Chamaedorea tepejilote* is a common species in the understory of tropical wet forests (Hodel 1992) and can be found in a wide range of locations and elevations (Oyama 1990). Our ENM results show that this species can live in a large part of the LCA territory. This likely maintains large effective population sizes, which reduce the effects of drift and maintain high levels of genetic diversity (Hamrick 1982; Hamrick *et al.* 1992). Greater genetic diversity is also expected in obligate outcrossing plants, such as dioecious species, which are not subjected to the inbreeding effects related to increasing homozygosity caused by selfing and geitonogamy (Glémin *et al.* 2006). The latter is supported by the lack of inbreeding observed in all studied populations of *C. tepejilote*.

Our findings indicate that the genetic structure of *C. tepejilote* populations estimated using SSRs can be attributed primarily to isolation between the Caribbean and Pacific slopes and secondarily to limited gene flow within slopes. A significant component structuring the studied populations of *C. tepejilote* are topographic barriers. Mountain ranges in Costa Rica divide the country from north-west to south-east, punctuated by small intermediate low-elevation passes among mountains, which are more common in the northern part of the country (Gabb 2007). The distribution of palm species is strongly determined by habitat characteristics such as temperature, humidity and precipitation (Eiserhardt *et al.* 2011) and on a continental scale, palm density and diversity are greater at lower elevations (Svenning 2001). Thus, climatic conditions found at high elevations are likely to act as a physiological barrier for lowland tropical species, particularly palms (Janzen 1967). In Costa Rica, palm density declines with increasing elevation (Lieberman *et al.* 1996) and they rarely occur above 2200 m a.s.l. (Hodel 1992). For *Chamaedorea*, climatic conditions at high elevations and at mountain passes impose a physiological barrier that limits population establishment and gene flow. Similar results have been shown for palm species in South America, where the Andean mountains act as an important barrier for mid-elevation palm species (Trénel *et al.* 2008). Thus, our results support our initial hypothesis that the Talamanca Mountain range would act as a barrier to gene flow between populations from the Pacific and Caribbean slopes.

We initially expected low levels of genetic structure among populations on the same slope estimated with SSR markers due to high levels of gene flow previously observed in other *Chamaedorea* species (Luna *et al.* 2005, 2007). However, our results contradict our initial predictions, as we found significant genetic structure among populations within the Caribbean and Pacific slopes. *Chamaedorea tepejilote* is pollinated both by wind and thrips (Ríos *et al.* 2013), which have been previously proposed as short-distance pollen dispersers (Cibrian-Jaramillo *et al.* 2009). Limited pollen dispersal may restrict gene flow among populations, resulting in significant differences in allele frequencies. We propose that the observed levels of genetic structure are also likely the result of recent changes in the landscape, as habitat loss and fragmentation may limit gene flow among populations, resulting in significant structure within slopes. *Chamadorea tepejilote* can establish large populations in old secondary forests and riparian habitats and is often found in protected areas along rivers (Ley-López and Avalos 2017). However, the recent decline in habitat availability due to forest fragmentation (Montero *et al.* 2021) may have reduced connectivity and gene flow between populations, resulting in increased population structure (Cristóbal-Pérez *et al.* 2021). Carvalho *et al.* (2015) and Peñaloza-Ramírez *et al.* (2016) found similar results for understory tropical palms, indicating that genetic differentiation is likely caused by recent habitat loss and fragmentation, and that these changes in landscape have a significant influence on genetic structure but not on intrapopulation variation. For two species of Andean *Chamaedorea*, Svenning (1998) found that their abundances increased in human-disturbed forests, suggesting that fragmentation may not necessarily reduce population sizes and that intrapopulation genetic diversity may be maintained. Genetic structure may be strongly influenced by landscape environmental features that, in conjunction with local adaptation (Brancalion *et al.* 2018; Coelho *et al.* 2020), may have a significant impact on the development of genetic structure among populations.

### Chloroplast DNA

Contrary to our initial expectations, we found little to no haplotypic diversity within populations. However, structure among populations was significantly greater for cpDNA markers than for SSRs, and our data did not support the hypothesis that mountains acted as barriers to gene flow. We originally hypothesized that during glacial oscillations, *C. tepejilote* may have survived in lowland refugia (Poelchau and Hamrick 2012) as these sites may have received enough precipitation and experienced less severe temperatures during the drier glacial periods of the Pleistocene (Hillesheim *et al.* 2005; Piperno 2006). These refugia would have maintained effective population sizes large enough to sustain high levels of genetic diversity (Petit *et al.* 2003), resulting in greater haplotype diversity in lowland populations. However, our cpDNA results did not support this hypothesis because haplotypic diversity within populations was almost non-existent, and populations were fixed for different haplotypes. Our analyses revealed significant phylogeographic structure (*N*_ST_ > *G*_ST_ for cytoplasmic markers), indicating that when populations were isolated, drift and mutation were more likely to influence structure than gene flow (Pons and Petit 1996). Our results do not support the hypothesis of a large lowland refugium; rather, as proposed by Brown (1987), our data indicate that several lowland and mid-elevation sites may have supported *C. tepejilote* populations during drier glacial periods, as previously suggested for other LCA plant species (Dick *et al.* 2003; Dick and Heuertz 2008; Jones *et al.* 2013). Alternatively, individuals of *C. tepejilote* may have persisted in fragments of humid riparian habitats that sustained vegetation during colder and drier periods (Meave and Kellman 1994). As with many other palm species, *C. tepejilote* is sensitive to drier environments, and during climate change, populations may have receded into smaller, isolated fragments of riparian habitat with enough humidity to sustain viable populations. Reduced population size and isolation may have increased the effects of drift, resulting in the fixation of distinct haplotypes among populations.

Our cpDNA analysis also showed that haplotypes are shared between Caribbean populations and populations found on the southern Pacific coast of Costa Rica (Figs 2 and 3), suggesting that gene flow may have occurred between these sites. In the southern part of Costa Rica, gene flow may have occurred south of the Talamanca Mountain range. When the Talamanca Mountain range reaches Panama, elevations drop significantly and habitat suitability for *C. tepejilote* extends along the Central Volcanic Chain of Panamá until the Darien depression (Fig. 4). It is likely that genes moved between the southern Caribbean coast and the southern Pacific coast through Panama. To our knowledge, this dispersal pattern has not previously been suggested for other plant species in LCA.

The elevation barrier imposed by high mountains, such as the Talamanca Mountain range with elevations over 3000 m a.s.l., has been considered a difficult barrier to overcome for animal and plant species that inhabit both sides of the cordillera (García-Rodríguez *et al.* 2021). Our SSR results show significant differences in allele frequencies between populations on both slopes **[see**Supporting Information—**Table S1****]**. These differences, however, were not observed for cpDNA markers. These results may be related to differences in pollen and seed dispersal in *C. tepejilote*, as well as differences in the mode of inheritance of SSR and cpDNA markers. Gene flow estimated using SSRs accounts for both seed and pollen dispersal, whereas cpDNA markers are uniparentally inherited and only reflect gene flow via seed dispersal. Our results suggest that mountain ranges may limit gene flow for thrips and wind. There is little information about the natural dispersal ability of thrips; however, studies in crops suggest that adult thrips dispersal is limited (Parker *et al.* 1991; Nyasani *et al.* 2017). Seed dispersal in *Chamaedorea* species occurs through frugivorous birds (Wheelwright *et al.* 1984), which are capable of overcoming topographic barriers moving between slopes and transporting seeds over long distances (Powell and Bjork 1994, 2004). Birds may disperse seeds through mountain passes or at the southern end of the Talamanca Mountain range, where lower elevations allow birds to move between slopes, reducing the structure between the Pacific and Caribbean populations. Additionally, dioecious species such as *C. tepejilote* may also have localized seed dispersal because only females can effectively disperse propagules, and dispersal occurs frequently in the vicinity of females, a phenomenon known as ‘seed-shadow handicap’ (Heilbuth *et al.* 2001; Barot *et al.* 2004). This would decrease local diversity and increase genetic structure among populations, especially for maternally inherited markers, as shown by our cpDNA results. Our results show that although mountain passes seem to be higher in the tropics (Janzen 1967), some species may simply go around them if they are given enough time or habitat is available on the outskirts of their range. Connectivity across the continental divide has important implications for conservation efforts, which should also consider low-elevation mountain passes as important conduits for gene flow. In these mountain passes, gene flow contributes to maintaining genetic diversity. However, a significant number of these mountain passes have lost habitat due to deforestation (Sader and Joyce 1988). Given the impending changes in precipitation and temperature brought on by climate change, it may be crucial to prioritize the conservation of these sites in order to maintain connectivity among populations.

We conclude that *C. tepejilote* is a widespread species in Costa Rica, with high levels of intrapopulation genetic diversity estimated using nuclear markers. Genetic structure patterns suggest that contemporary gene flow across the mountain ranges is restricted and the prevalent climatic conditions at high elevations represent a significant physiological barrier for this species. Our results also suggest that habitat loss and landscape changes have the potential to increase genetic structure if this species becomes isolated into small populations. During the dry cycles of the Pleistocene, riparian habitats and forest fragments may have played an important role as refugia for understory palms. Further research on the phylogeographic of LCA plants should focus on these areas to understand how these sites may have maintained high diversity with different life history traits and their potential as reservoirs of genetic diversity for tropical species.

## Supporting Information

The following additional information is available in the online version of this article—

**Figure S1.** Structure Harvester results for different values of *K*. (A) Delta *K* value estimated by the Evanno method. (B) Mean (± SD) of the estimated log of the probability of the data given *K* (Ln Pr(*X*|*K*)) estimated by the STRUCTURE program for 15 replicate runs for each *K* value. Red dots indicate the optimal *K* selected by each method.

**Figure S2.** Neighbour-joining tree based on pairwise *G*_ST_ values among 12 populations of *Chamaedorea tepejilote* (Arecaceae) in Costa Rica. See Table 1 for population abbreviations. Text color is based on *K* = 5 cluster assignment by STRUCTURE in Fig. 1. 

**Table S1.** Analysis of molecular variance (AMOVA) for SSR markers for populations of *Chamaedorea tepejilote* (Arecaceae) in Costa Rica, grouped in two regions: Pacific and Caribbean slopes.

**Table S2.** Analysis of molecular variance (AMOVA) using cpDNA haplotypes for populations of *Chamaedorea tepejilote* (Arecaceae) in Costa Rica, grouped into two regions: Pacific and Caribbean slopes.

plac060_suppl_Supplementary_Figure_S1

plac060_suppl_Supplementary_Figure_S2

plac060_suppl_Supplementary_Table_S1

plac060_suppl_Supplementary_Table_S2

## Data Availability

The data sets generated during the current study are available from https://doi.org/10.5281/zenodo.7337953.

## Notes

**Evolution & Diversity. Chief Editor:** Jeremy Beaulieu
