# Supplementary figures and images for: Genetic diversity and phylogeographic patterns of the dioecious palm Chamaedorea tepejilote (Arecaceae) in Costa Rica: the role of mountain ranges and possible refugia
Source: AoB Plants. 2022 Dec 17;15(1):plac060. doi: 10.1093/aobpla/plac060 (PMC9840212; doi:10.1093/aobpla/plac060)

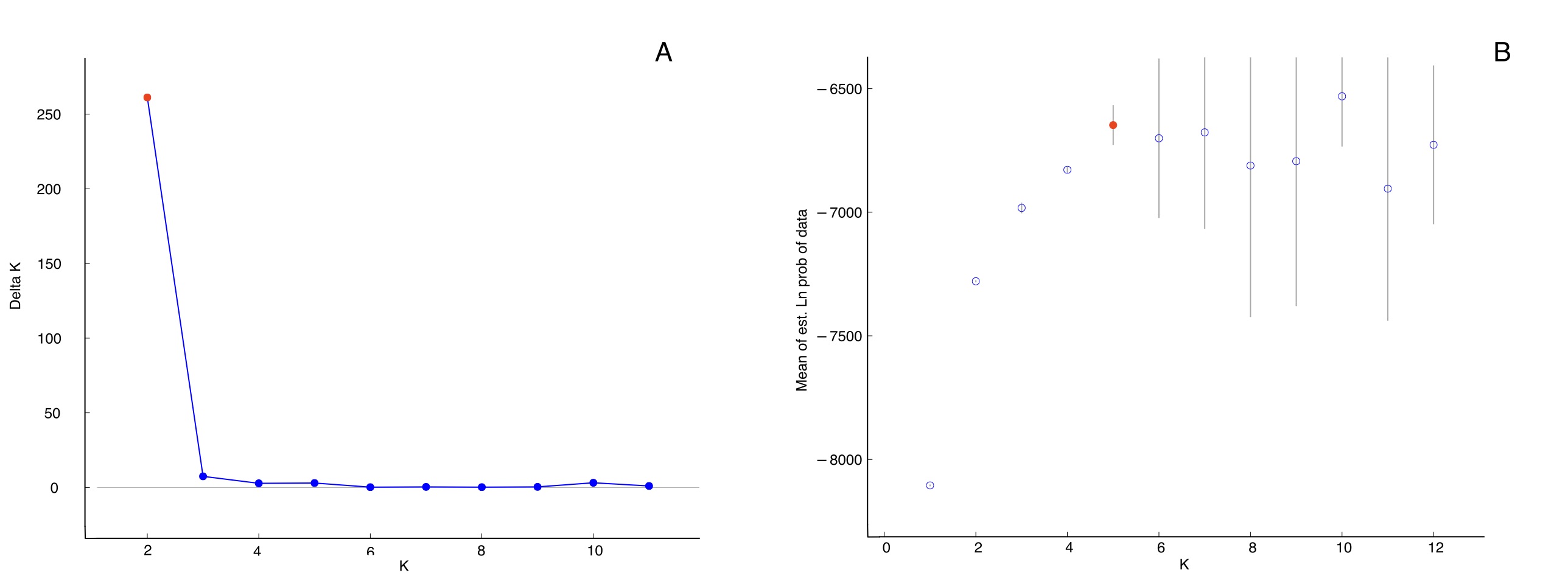

Supplement: plac060_suppl_Supplementary_Figure_S1 [file plac060_suppl_supplementary_figure_s1.jpeg]

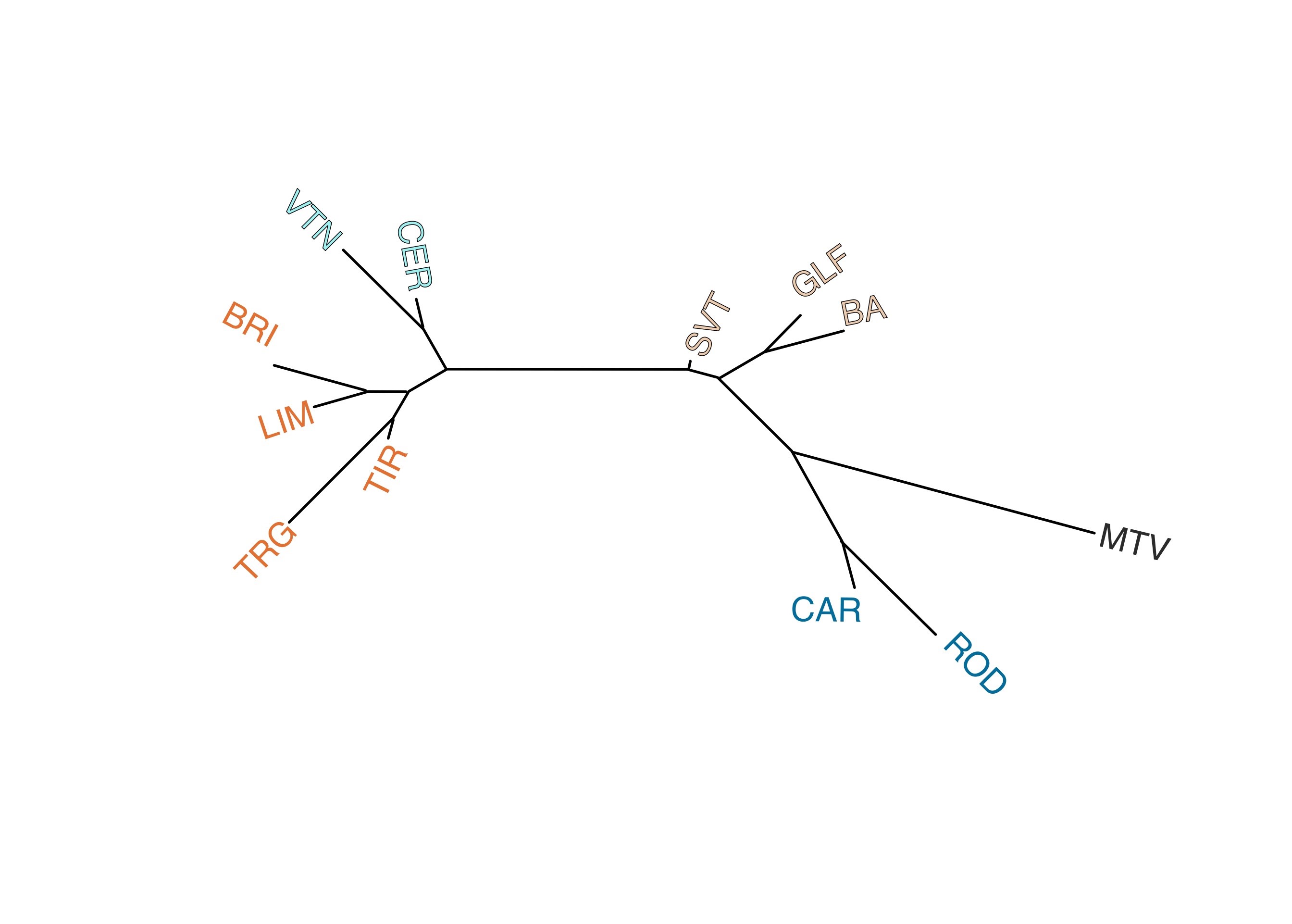

Supplement: plac060_suppl_Supplementary_Figure_S2 [file plac060_suppl_supplementary_figure_s2.jpeg]
