# Supplementary material for: Genetic diversity and phylogeographic patterns of the dioecious palm Chamaedorea tepejilote (Arecaceae) in Costa Rica: the role of mountain ranges and possible refugia
Source: AoB Plants. 2022 Dec 17;15(1):plac060. doi: 10.1093/aobpla/plac060 (PMC9840212; doi:10.1093/aobpla/plac060)
Supplement: plac060_suppl_Supplementary_Table_S1 [file plac060_suppl_supplementary_table_s1.docx]

Supplemental Table 1. Analysis of molecular variance (AMOVA) for SSR markers for populations of *Chamaedorea tepejilote* (Arecaceae) in Costa Rica, grouped in two regions: Pacific and Caribbean slopes.

| Source of Variation | df | Sum of Squares | Variance components | Percent of variation | Fixation indices |
| --- | --- | --- | --- | --- | --- |
| Between regions | 1 | 103.76 | 0.8502 | 17.77 | $\phi_{CT}$ = 0.178 *** |
| Among populations within regions | 10 | 160.60 | 0.7523 | 15.72 | $\phi_{SC}$ = 0.191 *** |
| Within populations | 57 | 617.24 | 3.1816 | 66.51 | $\phi_{ST}$ = 0.335 *** |
| Total | 205 | 881.596 | 4.7841 |  |  |

***: p < 0.001
