# Supplementary material for: Genetic diversity and phylogeographic patterns of the dioecious palm Chamaedorea tepejilote (Arecaceae) in Costa Rica: the role of mountain ranges and possible refugia
Source: AoB Plants. 2022 Dec 17;15(1):plac060. doi: 10.1093/aobpla/plac060 (PMC9840212; doi:10.1093/aobpla/plac060)
Supplement: plac060_suppl_Supplementary_Table_S2 [file plac060_suppl_supplementary_table_s2.docx]

Supplemental Table 2. Analysis of molecular variance (AMOVA) using cpDNA haplotypes for populations of *Chamaedorea tepejilote* (Arecaceae) in Costa Rica, grouped into two regions: Pacific and Caribbean slopes.

| Source of Variation | df | Sum of Squares | Variance components | Percent of variation | Fixation indices |
| --- | --- | --- | --- | --- | --- |
| Between regions | 1 | 24.23 | 0.2705 | 8.73 | $\phi_{CT}$ = 0.087 ^NS^ |
| Among populations within regions | 11 | 162.01 | 2.7251 | 87.96 | $\phi_{SC}$ = 0.964 *** |
| Within populations | 57 | 5.833 | 0.102 | 3.30 | $\phi_{ST}$ = 0.967 *** |
| Total | 69 | 192.27 | 3.098 |  |  |

^NS^: p>0.05; ***: p < 0.001
